# Comparative Evaluation of Harmless Acute Pancreatitis Score (HAPS) and Bedside Index of Severity in Acute Pancreatitis (BISAP) Scoring System in the Stratification of Prognosis in Acute Pancreatitis

**DOI:** 10.7759/cureus.32540

**Published:** 2022-12-15

**Authors:** Dimple Gupta, Nishith S Mandal, Jainendra K Arora, Rajesh K Soni

**Affiliations:** 1 Surgery, Vardhman Mahavir Medical College and Safdarjung Hospital, New Delhi, IND; 2 General Surgery, Vardhman Mahavir Medical College and Safdarjung Hospital, New Delhi, IND

**Keywords:** prognosis, bisap, haps, clinical investigations, acute pancreatitis

## Abstract

Acute pancreatitis is a common disease in patients presenting to the emergency room in any hospital. The most common causes are alcohol ingestion and gallstone disease. Diagnosis is usually based on clinical findings and elevated serum amylase and lipase levels. Imaging is often not necessary but may be used to confirm the diagnosis or rule out any other pathology or to evaluate for any complications. The majority of patients will have a mild, self-limiting disease but others may develop a severe fulminant course with organ failure. These patients are at high risk of developing complications, morbidity or mortality. Treatment of acute pancreatitis includes supportive treatment with antibiotics, fluids, analgesics and early enteral feeding. Several scores have been developed to predict the course of pancreatitis and help make informed decisions, monitoring and timely intervention. The majority of them are complicated, require extensive and expensive interventions or require time. Harmless acute pancreatitis score (HAPS) is one such score that is easy to calculate and is done at the time of admission, bedside index of severity in acute pancreatitis (BISAP) is another one requiring more parameters. The parameters used to calculate it are easily available and can be done at a majority of healthcare facilities in developing countries. HAPS thus seems to be a good option in aiding doctors in assessing acute pancreatitis. It may be considered as a standard scoring for acute pancreatitis for early and effective management. We have tried to study and compare the superiority of HAPS over BISAP in predicting prognosis in acute pancreatitis.

## Introduction

Acute pancreatitis (AP) is a common cause of acute abdominal pain in patients presenting in the emergency department. AP should be suspected in a patient with a history of biliary tract disease or alcohol abuse presenting with abdominal pain in the epigastrium radiating to the back, vomiting and distension of the abdomen [1].

Serum amylase and lipase have been used as biochemical markers to diagnose AP for many decades [2]. Imaging studies such as ultrasonography (USG) and computed tomography (CT) are confirmatory. Although many patients are managed successfully by medical management, 15%-20% of them develop complications that put them at significant risk of mortality. Reliable scoring systems, radiological evaluation and laboratory markers are required for identifying high-risk patients at an early stage in order to take prophylactic measures [3]. Several prognostic scoring systems like CT index, APACHE II, Ranson, etc. have been used to predict severity in AP but they are too complicated, too expensive and not available early in the course of the disease and not easily available at primary health care centres [1].

Harmless AP score (HAPS) is an easy to perform scoring system for AP and decides with great accuracy, which patients will run a mild course of pancreatitis [3]. HAPS contains three parameters - signs of peritonitis, levels of serum creatinine and haematocrit [4]. The patient is classified as HAPS negative (-) if they have an absence of signs of peritonitis, serum creatinine levels < 2 mg/dL, and haematocrit levels of <43% for males and <39.6% for females at the time of admission [1]. The patient is considered HAPS positive (+) if any one of the above parameters is positive. This score helps to identify the patients, who will have a non-severe course of AP, and who do not require intensive management and expensive imaging procedures.

Another simple prognostic scoring system was proposed by Wu et al. in 2008 for the early determination of the severity of AP, which they named as Bedside index of severity in AP (BISAP) [5,6]. BISAP considers five parameters - blood urea nitrogen >25 mg/dL, impaired mental status, presence of systemic inflammatory response syndrome (SIRS), age >60 years, and detection of pleural effusion by imaging [7]. For each parameter, one point is allotted, and the maximum total score is 5. A score of less than 3 is considered mild and more than or equal to 3 is considered severe pancreatitis. Early identification of patients at risk of developing severe pancreatitis or its complications will help us in triaging the patients and providing appropriate treatment.

## Materials and methods

This was a prospective observational cohort study conducted in the department of surgery at Vardhman Mahavir medical college and Safdarjung Hospital, New Delhi, India. The study was approved by the Safdarjung hospital Institutional ethics committee vide approval number: SJH/IEC/Surgery/PG-2018.

Sixty patients of AP presenting to the surgical emergency room with the first attack of AP during the period from October 2018 to March 2021 were studied. A comparative study of HAPS and BISAP scores was done in all the patients.

Inclusion criteria

All patients of more than 12 years of age, clinically diagnosed as having AP presenting to the surgical department were included in the study.

Exclusion criteria

Any patient with traumatic pancreatitis, post-operative pancreatitis, malignancy, post-ERCP pancreatitis, or pregnant or immunocompromised patients was excluded from this study.

Sample size:

The study by Talukdar et al. [5] observed sensitivity and specificity of the BISAP score were 71.4% and 99.1%, and of HAPS was 76.3% and 85.7%. Taking these values as a reference, the minimum required sample size with desired precision of 15%, 80% power of study and 5% level of significance is 62 patients. So total sample size taken was 60.

Sample size calculation

H0: Se = 71.4 versus Se ≠ 71.4 (Se 1)

With 95% confidence level and 80% power for detection of difference of 15% from a Se of 71.4%, sample size calculated is:

N = ((1.96 * sqrt (0.714 * (1 - 0.714)) + (0.84 * sqrt (0.839 * (1 - 0.839))2/(0.15 * 0.15) = 61.22 = 62 (approx.)

H0: Se = 76.3 versus Se ≠ 76.3 (Se 1)

With 95% confidence level and 80% power for detection of difference of 15% from a Se of 76.3%, sample size calculated is:

N = ((1.96 * sqrt (0.763 * (1 - 0.763)) + (0.84 * sqrt (0.913 * (1.913)) 2 / (0.15 * 0.15) = 50.90 = 51 (approx.)

Study design

On admission, a detailed history of the patient was taken based as per the proforma and patient was thoroughly examined and investigated. After this evaluation, patients having AP were enrolled in the study. The HAP score and BISAP score were calculated within one hour of admission.

HAP score includes signs of peritonitis, haematocrit level (abnormal when more than 43% for men and more than 39.6 % for women) and serum creatinine level (abnormal when more than 2mg/dL). Each variable is assigned one point and the total score ranges from 0 to 3. 

BISAP Score includes blood urea nitrogen (abnormal when more than 25mg/dL), impaired mental status, SIRS, age (abnormal when age more than 60 years) and presence of pleural effusion as detected on imaging. Each variable is assigned one point each and the total score ranges from 0 to 5.

Standard treatment of AP was started in all the patients and patient were observed for development of local and/or systemic complications. Appropriate treatment was given for the complications. Patient was assessed on the basis of BISAP score and HAP score and evaluated.

Statistical analysis

Categorical variables were presented in number and percentage (%) and continuous variables were presented as mean ± SD and median. Diagnostic test was used to calculate sensitivity, specificity, PPV and NPV. Inter-rater kappa agreement was used to find out the strength of agreement between HAPS and BISAP with outcome. Mcnamer test was used to compare sensitivity and specificity. A comparison of receiver operating characteristic curve was used to compare area under curve of HAPS and BISAP for predicting outcome. A p-value of <0.05 was considered statistically significant. The data were entered in MS EXCEL spreadsheet and analysis was done using Statistical Package for Social Sciences (SPSS) version 21.0.

## Results

The study group of 60 patients was selected and the HAPS and BISAP scores of the patients were calculated on the first day of admission and the first attack of AP. Pancreatitis can affect any age group and in the present study of 60 patients, most of the patients fell under the age group of 31 to 50 years of age. There was a female preponderance in our study with a female distribution of 37 (61.67%) and male distribution of 23 (38.33%).

In our study, signs of peritonitis were present in 21 (35.00%) patients and absent in the rest. Out of the 21 patients with signs of peritonitis, 11 (52.38%) of them developed severe AP. According to HAPS, the presence of abdominal guarding/rigidity is associated with a severe course of AP.

In our study, serum creatinine values (≥ 2 mg/dL) were raised in five out of 60 patients (8%) of the patients only. However, 11 out of the 60 patients developed severe AP. It has been observed in our study that higher values of serum creatinine, at the time of admission are associated with a severe course of pancreatitis and the development of complications like persistent organ failure.

In our study, haematocrit values were raised in only seven (30.43%) males and seven (18.91%) females. Out of the seven male patients, three developed severe AP, while in the female group eight patients developed severe AP. An admission haematocrit ≥44% or failure of the haematocrit to decrease 24 hours following admission, is indicative of severe AP in the early stage of the disease.

In our study, pleural effusion was present in 29 (48.33%) patients. Out of these, 11 (37.93%) patients developed severe AP. Those who did not have pleural effusion did not develop severe AP in the course of their hospital stay. According to the BISAP score, the presence of pleural effusion is one of the indicators of developing a severe course of AP.

**Figure 1:**
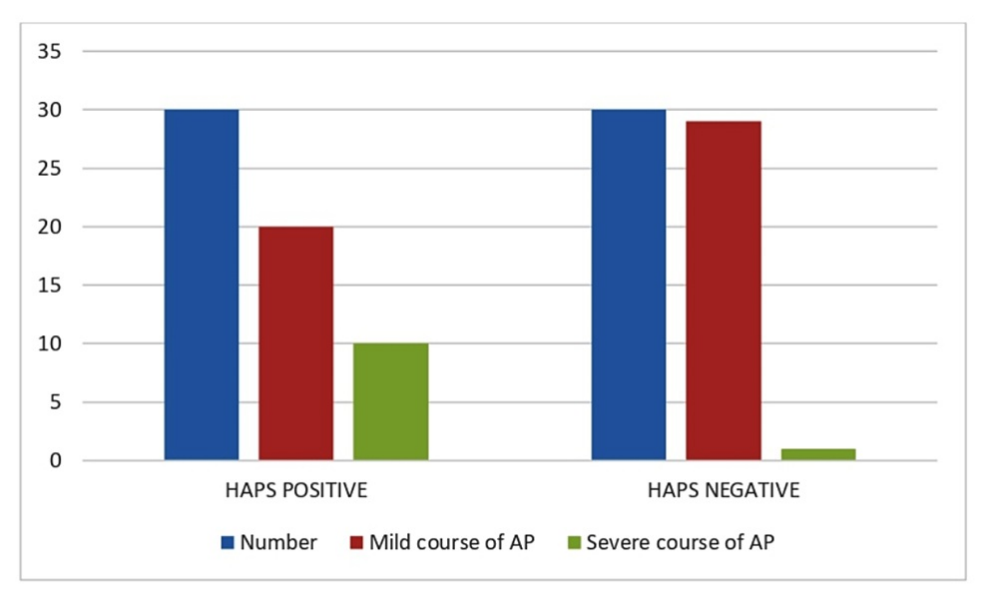
Distribution of outcome of HAPS assessment of patients

In our study, only four (6.67%) patients showed signs of SIRS. However, all of them developed severe AP. BISAP predicts that the presentation of patients with SIRS is associated with a severe course of AP. In our study, only two (3.33%) subjects showed impaired mental status. However, 11 patients ultimately developed severe AP. BISAP score predicts that a patient with impaired mental status has a higher likelihood of developing a severe AP. The distribution of the outcome of the HAPS assessment of study subjects is shown in Figure 1. 

**Figure 2:**
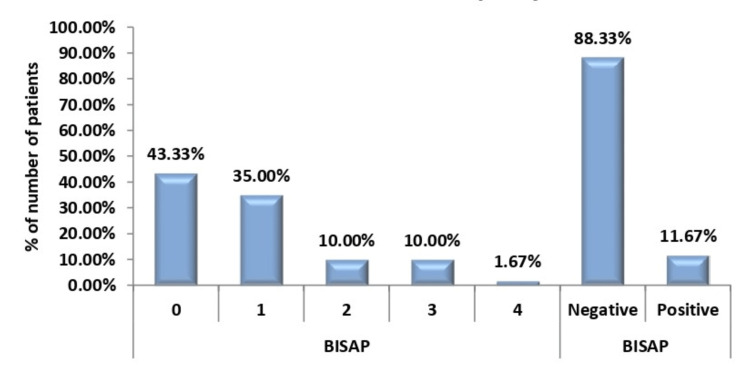
Distribution of outcome of BISAP assessment of patients

The distribution of the outcome of the BISAP assessment of study subjects is shown in Figure 2.

## Discussion

AP can occur in any age group, but middle age is most commonly affected, in a similar study by Machicado et al. [8]. Studies by Ong et al. and Corfield et al. also support our findings that advanced age is associated with a higher mortality rate [9,10]. There is a female preponderance of cases of AP as compared to males, which is also seen in studies by Roberts et al. and Yadav et al. [11,12]. There are studies by Wan et al., Muddana et al. and Lankisch et al. that concluded that a serum creatinine level > 1.8 mg/dL within 24 hours of admission can positively predict persistent organ failure in AP and that values < 1.8 mg/dL can be useful for negatively predicting organ failure in AP, which is similar to the observations seen in our study [13,14,15]. Haematocrit > 44% at the time of admission or the failure of haematocrit to decrease within 24 hours after admission is associated with an increased risk of developing necrotizing AP while the absence of haemo-concentration has a higher negative predictive value (NPV) for the development of severe AP, in similar studies by Berger et al., Lankisch et al. and Gardner et al. [16-18]. Studies by Lankisch et al. have shown that chest x-ray with pleural effusion is one of the indicators for developing severe AP, as in our study [19,20]. BISAP score has shown that patients with SIRS and impaired mental status have a higher likelihood of developing severe AP, similar findings have been reported by Singh et al. and Mofidi et al. [21,22]. A study of Balthazar classification by Gulen et al. has suggested that neutrophil/lymphocyte ratio and RDW were not effective in determining mortality in patients with AP [23].

In our study, 30 subjects (50% of the patients) with AP were HAPS positive, while the rest 30 of them (50%) were negative, which means that 50% of our patients were predicted to have a severe disease while 50% were predicted to have a mild course. Out of those 30 that were HAPS positive (predicted to run a severe course), 11 patients out of the 30 (36.66% of the 50% which were HAPS positive) actually had a severe course during the course of their hospital stay, while seven (out of those 11) patients expired. Out of 30 who were HAPS negative, only one (3.33%) eventually developed severe AP. The sensitivity of HAPS as per our study in predicting severe AP is 90.91%, while specificity is 59.18% only. Thus, HAPS has a higher sensitivity while predicting mortality and the course of severity, similar to a study by Oskarsson et al. [24]. The diagnostic accuracy of HAPS stands at around 65%. However, the NPV is 96.67%. So, if HAPS is negative the patient will usually run a mild course. However, if the score is positive, the patient can run in either direction, like the study by Qahtani et al. [25]. Thus, HAPS helps to decide, if any patient will require expensive imaging procedures and saves substantial hospital costs. The high NPV states that the HAPS scoring system is capable of identifying with great accuracy, patients who will run a mild course and do not require intensive management and who are not in danger of dying of the disease, within the first hour of admission. Thus, the HAPS scoring system helps to efficiently filter out the majority of the bulk of patients with AP who need not be managed overzealously, similar to the study by Ma et al. [26].

In our study, the BISAP score was less than three for 57 (88.33%) study subjects and was equal to or more than three for seven (11.67%) patients. The majority of patients had a score of zero on presentation (26 patients, 43.33%). Out of the patients predicted by BISAP with a severe course of AP, all of them developed severe AP while the majority of them expired during their stay in the hospital, like the study by Arif et al. [27]. A score of 3 or more than 3 in BISAP is predictive of poor prognosis and a high chance of running a severe course of pancreatitis, as per a study by Kaushik et al. [28]. Thus, BISAP has a sensitivity of 63.64% and high specificity of 100%, like the study by Venkatapuram et al. [29]. The diagnostic accuracy of BISAP is also high, at around 93%. However, the diagnostic accuracy of HAPS stands at around 65%. The sensitivity, specificity, and diagnostic accuracy of HAPS and BISAP are shown in Table 1.

**Table 1:** Sensitivity, specificity, positive predictive value and negative predictive value of HAPS and BISAP for predicting severe acute pancreatitis CI: Confidence interval; AUC: Area under curve.

Severe acute pancreatitis	HAPS	BISAP
Sensitivity (95% CI)	90.91% (58.72% to 99.77%)	63.64% (30.79% to 89.07%)
Specificity (95% CI)	59.18% (44.21% to 73.00%)	100% (92.75% to 100.00%)
AUC (95% CI)	0.75 (0.62 to 0.85)	0.82 (0.70 to 0.91)
Positive Predictive Value (95% CI)	33.33% (17.29% to 52.81%)	100% (59.04% to 100.00%)
Negative Predictive Value (95% CI)	96.67% (82.78% to 99.92%)	92.45% (81.79% to 97.91%)
Diagnostic accuracy	65.00%	93.33%
Kappa	0.3	0.741
P value of Kappa	0.003	< .0001>

**Figure 3:**
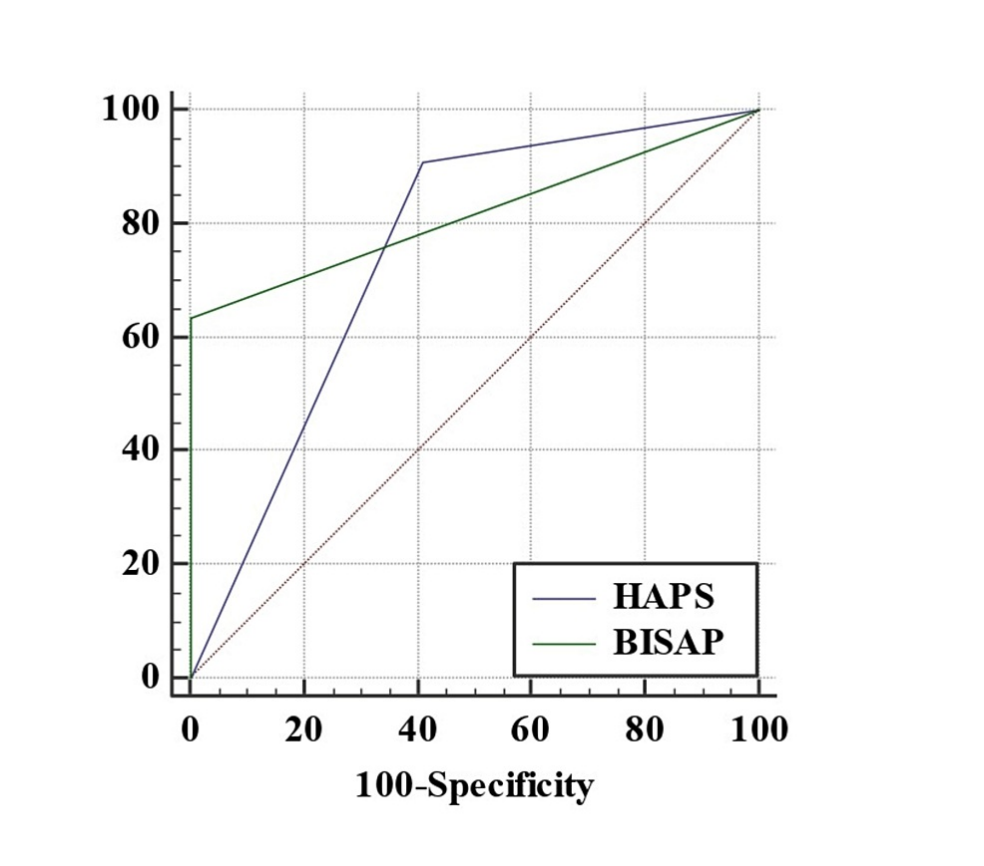
Comparison of area under curve of HAPS and BISAP for predicting severe acute pancreatitis

So, our present study shows that both the scores, HAPS and BISAP can be used to predict the prognosis of the course of AP in the patient. The area under curve, sensitivity and specificity of HAPS and BISAP is shown in Figure 3.

The K (Kappa) value can be interpreted in terms of strength of agreement (SA) with our findings. If the K-value is < 0.20 , then the SA is poor. If the K0value is between 0.21 and 0.40, then the SA is fair. The K-value for HAPS is 0.3, which amounts to fair agreement with our study findings. If the K-value is from 0.41 to 0.60, then the SA is moderate. If the K-value is between 0.61 and 0.80, then the SA is good. The K-value for BISAP is 0.741, which is in good agreement with our study. If the K-value is from 0.81 to 1.00, then the SA is very good. It may be concluded that BISAP is a better predictor of prognosis for AP as compared to HAPS. The results are shown in Table 2. 

**Table 2:** Difference between area under curve, sensitivity and specificity of HAPS and BISAP for predicting severe acute pancreatitis AUC: Area under curve.

Severe acute pancreatitis		HAPS vs BISAP
Difference between areas		0.0677
Standard Error		0.104
95% Confidence Interval		-0.136 to 0.271
P value	AUC	0.5139
	Sensitivity	0.375
	Specificity	< .0001>

Limitations of our study

Since our study was a single-arm study, the results of our study could not be compared with a direct concurrent group. Our study sample was small with only 60 patients. Single-centre studies have the disadvantage that they usually are conducted by a highly motivated researcher. Thus, the findings may be more positive than they would be in reality. Since our study was based on patients admitted with gallstone-induced pancreatitis, the findings may be variable in patients admitted with other causes of pancreatitis. Also, as the study of HAPS is on patients presenting for the first time with AP, we could not study the patients for recurrence of the attack.

## Conclusions

 

Several scores have been developed to predict the course of AP and help make informed decisions, monitoring and timely intervention. The majority of them are complicated, require extensive and expensive interventions and are time-consuming. However, the vast majority of patients of AP experience a milder course of the disease. So, it is more important to identify the mild cases of AP, wherein HAPS could have significant utility as compared to other scoring systems in triaging patients with AP. HAPS is one such score which is easy to calculate and is routinely done at the time of admission of the patients. The parameters used to calculate it are easily available and can be done at the majority of healthcare facilities in developing countries. As per our study, it was found that HAPS is a highly sensitive score, having 90.91% sensitivity when it comes to predicting the severity of the course of AP. It was also concluded that, if HAPS is negative, then the patient is more likely to run a mild course of AP, i.e., it has a very high NPV of 96.69%. Also, it helps to make a decision as to which patient might require intensive monitoring. HAPS thus seems to be a good option in aiding doctors in assessing the severity of AP. HAPS may be considered a gold standard for the prognostication of AP for early and cost-effective management.
